# SARS-CoV-2 Remains Infectious on Refrigerated Deli Food, Meats, and Fresh Produce for up to 21 Days

**DOI:** 10.3390/foods11030286

**Published:** 2022-01-21

**Authors:** Mo Jia, Tina M. Taylor, Sterling M. Senger, Reza Ovissipour, Andrea S. Bertke

**Affiliations:** 1 Population Health Sciences, Virginia Maryland College of Veterinary Medicine, Virginia Polytechnic Institute & State University, Blacksburg, VA 24061, USA; moj@vt.edu (M.J.); ttina@vt.edu (T.M.T.); 2 Food Science and Technology, Agricultural Research and Extension Center, Virginia Polytechnic Institute & State University, Hampton, VA 23669, USA; sterlingms@vt.edu (S.M.S.); ovissi@vt.edu (R.O.); 3 Center for Emerging Zoonotic and Arthropod-Borne Pathogens, Virginia Polytechnic Institute & State University, Blacksburg, VA 24061, USA

**Keywords:** SARS-CoV-2, COVID-19, foodborne transmission, food contamination, plaque assay, qRT-PCR, thermal processing, cooking

## Abstract

SARS-CoV-2, the virus that causes COVID-19, has been detected on foods and food packaging and the virus can infect oral cavity and intestinal cells, suggesting that infection could potentially occur following ingestion of virus-contaminated foods. To determine the relative risk of infection from different types of foods, we assessed survival of SARS-CoV-2 on refrigerated ready-to-eat deli items, fresh produce, and meats (including seafood). Deli items and meats with high protein, fat, and moisture maintained infectivity of SARS-CoV-2 for up to 21 days. However, processed meat, such as salami, and some fresh produce exhibited antiviral effects. SARS-CoV-2 also remained infectious in ground beef cooked rare or medium, but not well-done. Although infectious SARS-CoV-2 was inactivated on the foods over time, viral RNA was not degraded in similar trends, regardless of food type; thus, PCR-based assays for detection of pathogens on foods only indicate the presence of viral RNA, but do not correlate with presence or quantity of infectious virus. The survival and high recovery of SARS-CoV-2 on certain foods support the possibility that food contaminated with SARS-CoV-2 could potentially be a source of infection, highlighting the importance of proper food handling and cooking to inactivate any contaminating virus prior to consumption.

## 1. Introduction

Since the coronavirus disease 2019 (COVID-19) pandemic began in late 2019, more than 341 million cases and 5.5 million deaths have been reported worldwide, as of January 2022 [1]. The causative agent of the pandemic, severe acute respiratory syndrome coronavirus 2 (SARS-CoV-2), is an enveloped, single-stranded, negative-sense RNA virus classified to the family *Coronaviridae*, genus *Betacoronavirus* [2]. SARS-CoV-2 is known to be transmitted person-to-person, primarily by airborne transmission and respiratory droplets (e.g., coughing, sneezing, talking) [3,4,5,6,7]. However, alternative routes of transmission have not been adequately investigated.

In addition to respiratory disease, COVID-19 patients also develop gastrointestinal symptoms, including nausea, vomiting, abdominal pain, and diarrhea [8,9,10]. Approximately half of COVID-19 patients shed the virus in feces, which persists longer than in respiratory secretions [11,12,13]. In some cases, the GI symptoms precede or even preclude respiratory disease [14]. Similar to other closely related coronaviruses, SARS-CoV-2 mediates viral entry into host cells by binding to the angiotensin-converting enzyme 2 (ACE2), which is expressed in respiratory epithelial cells [15,16,17]. However, the ACE2 receptor is also highly expressed in the oral cavity, salivary glands, and gastrointestinal tract (GI) tissues, such as stomach, cholangiocytes, enterocytes from the ileum and colon, and rectum cells [18,19]. SARS-CoV-2 has been shown to infect cells in the oral cavity, the salivary glands, enterocytes and intestinal organoids [13,19,20,21]. In Rhesus macaques, intragastric inoculation with SARS-CoV-2 resulted in the infiltration of inflammatory cells and loss of mucosal epithelium in gastrointestinal tissues [20]. A decreased number of goblet cells was observed in the small intestine as early as 1 day post-inoculation (dpi), highlighting the apoptosis of gastrointestinal epithelial cells caused by SARS-CoV-2 infection [20]. Decreases in goblet cell numbers in COVID-19 patients have also been reported, indicating that the induced intestinal inflammation by SARS-CoV-2 also occurs in humans [22]. Infection of human oral epithelial cells and salivary glands was confirmed, with a salivary viral burden correlating with COVID-19 symptoms [19]. Taken together, a growing collection of evidence suggests that SARS-CoV-2 may be able to infect the host via ingestion through the GI route.

The World Health Organization (WHO), US Food and Drug Administration (FDA), and European Food Safety Authority (EFSA) have issued statements that food is unlikely to be associated with transmission of SARS-CoV-2 [23,24,25]. However, reports of SARS-CoV-2-contaminated foods and food packaging raise concerns about potential health risks associated with food, food packaging, and food contact surfaces that may become contaminated during processing, transport, or preparation [26,27,28,29,30]. The low-temperature environments during storage and transportation of foods support favorable conditions for the survival of SARS-CoV-2. Several studies have shown that SARS-CoV-2 can survive on certain types of foods, including seafood, meat, and produce under refrigeration (4 °C) and frozen (−10 °C to −80 °C) conditions, with the virus in some foods remaining infectious for more than 21 days [29,30,31]. In addition, outbreaks of COVID-19 have been linked to SARS-CoV-2-contaminated frozen food and food packaging through phylogenetic analysis of virus from patients and food or packaging materials [26,27]. On common food contact surfaces, infectious SARS-CoV-2 is stable on stainless steel and plastic for up to 72 h [32]. Taken together, these studies suggest that contamination of food and environmental surfaces by SARS-CoV-2 may present a potential risk of infection for susceptible individuals.

A complete understanding of transmission routes for any pathogen is essential to guide the development of appropriate public health policies and control measures to prevent, or at least reduce, the risk of infection and spread within the population. Given the mounting evidence that SARS-CoV-2 can infect cells of the oral cavity and GI tract, one must consider the possibility that infection can potentially occur through ingestion of virus-contaminated foods. Since some types of food may present a greater risk of infection than others, we sought to determine the relative risks of infection from various types of foods. We previously developed and validated methods to effectively recover infectious enveloped viruses from the surface of different types of food and investigate the survival of SARS-CoV-2 on a limited number of foods over a 24 h period of time (chicken, salmon, shrimp, spinach, apple skin, and mushroom) [29]. Others have focused on assessing the survival of SARS-CoV-2 on cold-chain seafoods and meats [26,27,28]. The objective of the current study was to identify additional types of foods that may present a potential risk of infection, by assessing the time of survival of SARS-CoV-2 on foods grouped into three broad categories, including ready-to-eat deli foods, fresh produce, and meats (which includes seafood), held at refrigeration temperature (4 °C) up to 21 days. Since ground beef burgers are commonly cooked rare or medium, instead of well done, we also assessed survival of SARS-CoV-2 in ground beef burgers cooked to various internal temperatures. Furthermore, since PCR-based assays are commonly used to detect pathogens on food and food packaging, we compared viral RNA quantities with infectious virus titers to determine if PCR-based assays could accurately assess the risk of infection presented by the presence of infectious virus. This study addresses knowledge gaps on understanding the potential of food products as carriers of SARS-CoV-2, particularly ready-to-eat foods and those that are consumed raw or intentionally under-cooked, such as ground beef burgers.

## 2. Materials and Methods

### 2.1. Cells and Viruses

SARS-CoV-2 (Isolate USA-WA1/2020, NR-52281), obtained from BEI Resources (Manassas, VA, USA), was propagated and titrated on Vero-E6 cells (ATCC CRL-1586). First passage virus stock was thawed on ice and serially diluted tenfold in Dulbecco’s Modified Eagle Medium (DMEM, Fisher Scientific, Waltham, MA, USA) for preparation of inocula.

### 2.2. Food Sources and Food Sample Preparation

Food samples were prepared based on the methods developed in our previous studies [29]. Fresh foods were purchased from a local grocery store for each experiment, maintaining identical brands for each replicate. Three broad categories of food types were selected in this study (Table 1): ready-to-eat deli foods (roasted turkey, cheese, and salami), fresh produce (tomato, avocado shell and pulp, and grape), and meats (beef steak, ground beef, pork chop, ground pork, plant-based meat alternative, and oyster). Turkey, cheese, salami, avocado shell and pulp, beef steak, pork chop, and oysters were cut to approximately 1.5 cm^2^ pieces and placed into sterile 12-well tissue culture plates for inoculation with virus. Approximately 1.7 g of ground beef, ground pork, and plant-based meat alternative were also placed into 12-well tissue culture plates for inoculation. To best mimic a real contamination of fresh produce during transportation or handling, whole grapes and cherry tomatoes were placed into screw-top specimen containers for inoculation. Both avocado shell and pulp were included in food samples, since the avocado shell is the standard presentation in the grocery store and avocado pulp is typically consumed raw.

### 2.3. Virus Inoculation on Foods

Virus inoculation and recovery were performed using methods similar to those developed in our previous studies [29], as we demonstrated that these methods achieved a high recovery rate close to 100%. Food samples were inoculated with 1 × 10^4^ PFU (20 µL) of SARS-CoV-2 in multiple small droplets (<1 to 2 µL per droplet) on the food surfaces, to best mimic deposition of virus contained in droplets from a cough or sneeze. To assess maximum virus recovery from each food type, the virus was recovered immediately after inoculation (0 h) by adding 1 mL DMEM and rinsing the food surface five times by pipette. Additional food samples were inoculated and stored at refrigeration temperature (4 °C) in sealed containers to prevent desiccation. At the designated time points (1 hour (1 h), 24 h, 7 days (7 d), 14 d, and 21 d post-inoculation), the virus was recovered by rinsing with 1 mL of DMEM five times using a pipette to maximize virus recovery. Rinse fluid was collected in microcentrifuge tubes and stored at –80 °C until titer was quantified by plaque assay. For negative control samples, uninoculated food samples were rinsed and stored using the same methods.

### 2.4. Ground Beef Cooking

Ground beef (25 g) was inoculated in a Whirl-Pak^®^ bag (Madison, WI, USA) with 1 × 10^4^ PFU/g of SARS-CoV-2. The inoculated ground beef was massaged by hand in the sealed bag for 2 min to thoroughly mix the virus into the matrix of the meat, placed into a 4 °C refrigerator for 10 min to allow for virus spreading within the meat, followed by dividing and forming the ground beef into 1.25 cm thick patties. Ground beef patties were then cooked on a clamshell-style electric grill (George Foreman model GRS040BC, Beachwood, OH, USA) to an internal temperature of 125 °F/51.2 °C (rare), 145 °F/62.5 °C (medium), or 160 °F/71.1 °C (well-done). Internal temperature of the beef patties was monitored via a Habor^®^ digital thermometer (Habor Precision Inc., Taichung City, Taiwan). When the designated temperature was reached, cooking time was recorded for each cooked sample and beef patties were immediately cooled by placing the patty into a Whirl-Pak^®^ bag and adding 20 mL of pre-chilled DMEM to prevent further cooking, followed by a thorough massage by hand to detach any virus that may have bound to the beef patties into the media. The rinse media (1 mL, in duplicate) from each Whirl-Pak^®^ bag was transferred into microcentrifuge tubes and stored at −80 °C until titration by standard plaque assay. As a negative control, uninoculated food samples were processed identically.

### 2.5. Plaque Assay

A standard plaque assay was performed on each sample. Virus samples were serially diluted tenfold with DMEM. Diluted virus samples were inoculated onto confluent Vero E6 monolayer cells in 24-well plates in duplicate. The infected plates were incubated for 1 h at 37 °C with 5% CO_2_ and then inoculum/dilutions were replaced with an agarose overlay consisting of DMEM supplemented with 8% Fetal Bovine Serum (ThermoFisher, Waltham, MA, USA), 1% penicillin-streptomycin (ThermoFisher, Waltham, MA, USA), and 0.5% molecular grade agarose (MedSupply Partners, Atlanta, GA, USA). Back-titration of the inoculum (in triplicate) was also performed to verify the inoculum concentration. Infected plates with agarose overlay were incubated for 48 h at 37 °C with 5% CO_2_, and then fixed with 37% formaldehyde and stained with plaque dye. Concentration of infectious virus was enumerated by plaque counts, and the final concentrations were expressed as plaque-forming units per mL (PFU/mL).

### 2.6. qRT-PCR

To extract SARS-CoV-2 RNA, 200 µL of food rinsing fluid from each food sample was mixed with an equal volume of TRI Reagent LS (ThermoFisher, Waltham, MA, USA). Viral RNA was extracted and purified using Direct-zol^TM^ RNA microPrep kit (Zymo Research, Irvine, CA, USA). A NanoDrop 2000 spectrophotometer (ThermoFisher, Waltham, MA, USA) was used to measure concentrations and purity of the extracted viral RNA using reading absorbances at 260 nm and 280 nm. To determine RNA genome copy number, 10 µL qRT-PCR reactions were prepared using the iTaq Universal Probe One-Step Kit (BioRad, Hercules, CA, USA), mixed with primers and probes specific for SARS-CoV-2 N protein, and then amplified on a ViiA 7 Real-Time PCR System (Applied Biosystems, Foster City, CA, USA) [33]. The standard setting used for qRT-PCR is as follows: 1 cycle of 10 min at 50 °C followed by 2 min at 95 °C; and 45 cycles of 3 s at 95 °C and 30 s at 55 °C. Final results were reported as genome copy number per mL of food rinsing fluid, to allow for direct comparison to infectious viral titer measured in PFU/mL.

### 2.7. Statistical Analysis

All experiments were conducted three times in duplicate. Plaque assay data were converted to log PFU/mL prior to statistical analysis. Statistical analysis was performed using SAS version 9.4 (SAS Institute, Cary, NC, USA) using analysis of variance via the GLIMMIX procedure. Significant differences between means were determined based on the calculations of least square means at the level of *p* < 0.05.

## 3. Results

COVID-19 patients have been reported to generate 1.7 × 10^4^ copies of SARS-CoV-2 in sputum during a typical cough, which could be deposited on the surface of foods during processing or handling [34]. Therefore, to assess the survival of SARS-CoV-2 on the surface of food using a relevant inoculum dose, we inoculated the surface of food samples with approximately 1 × 10^4^ PFU in multiple droplets. The virus was recovered immediately after inoculation (0 h) to quantify the maximum recoverable virus from each food, and after five incubation times (1 h, 24 h, 7 d, 14 d, 21 d) at refrigeration temperature (4 °C), using a rinsing method for virus recovery that we previously validated for recovery of enveloped virus from food surfaces [29]. We assessed the survival of SARS-CoV-2 on thirteen foods, grouping them based on general categories of the foods.

### 3.1. Survival of SARS-CoV-2 on Deli Foods

**Figure 1:**
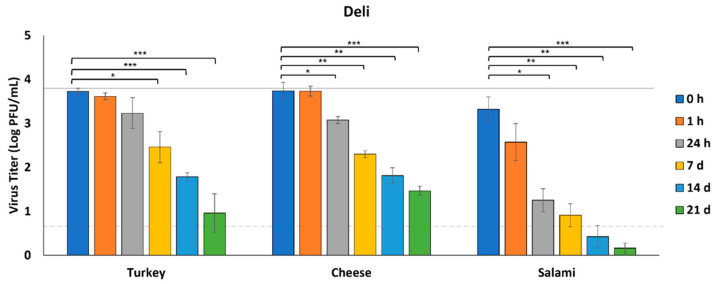
Survival of SARS-CoV-2 on deli foods. SARS-CoV-2 was recovered from foods immediately after inoculation (0 h) and at 1 h, 24 h, 7 days (d), 14 d, and 21 d after inoculation at 4 °C (*n* = 3). Infectious SARS-CoV-2 was quantified by plaque assay on Vero E6 cells and shown as log PFU/mL. The inoculum is shown as a solid gray line (3.9 log PFU/mL) and the detection limit of the plaque assay is shown as a dashed line (0.7 log PFU/mL). Significant differences between means are indicated by asterisks (* < 0.05; ** < 0.01; *** < 0.001).

We selected three types of food that represent general items commonly purchased from a grocery store delicatessen (deli), including roasted turkey representing minimally seasoned cooked meats, Swiss cheese representing cheeses in general, and salami representing a more processed and seasoned type of meat. We did not observe a significant reduction in virus titer recovered from turkey over the first 24 h (Figure 1). Although a significant reduction in virus concentration was observed at 7 and 14 days post-inoculation (dpi) (2.5 and 1.8 log PFU/mL, respectively, *p* < 0.05), infectious virus could still be recovered from the turkey at 21 dpi (1.0 log PFU/mL) (Figure 1). Virus concentration from cheese was significantly reduced by 24 h post-inoculation (3.7 log PFU/mL at 0 h and 3.1 log PFU/mL at 24 h, *p* < 0.05), and continued to decline over time. However, similar to turkey, a viable virus was still recovered at 21 dpi (1.5 log PFU/mL) (Figure 1). From salami, however, virus concentration was significantly reduced 2 log PFU/mL by 24 h post-inoculation (3.3 log PFU/mL at 0 h to 1.3 log PFU/mL at 24 h, *p* < 0.05) and to below the level of detection (0.7 log PFU/mL) by 14 dpi (Figure 1).

### 3.2. Survival of SARS-CoV-2 on Produce

We selected produce items that are typically consumed raw, including tomatoes, grapes, and avocados. To avoid any effects from the pulp or juice, whole cherry tomatoes and whole grapes were assessed for virus survival by inoculating SARS-CoV-2 on the surface of these fruits. Since avocados are cut to remove the pulp, which may introduce contamination from the avocado shell into the pulp, we assessed both outer avocado shell and avocado pulp for survival of the virus.

**Figure 2:**
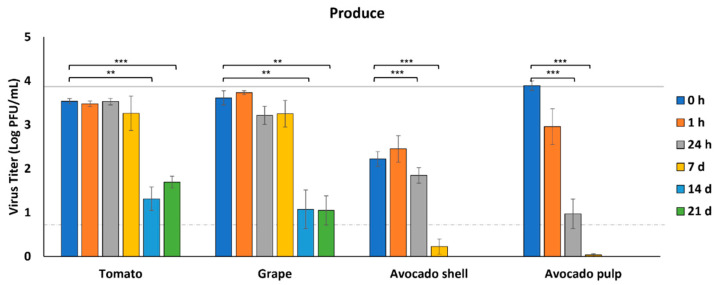
Survival of SARS-CoV-2 on fresh produce. SARS-CoV-2 was recovered from foods immediately after inoculation (0 h) and at 1 h, 24 h, 7 d, 14 d, and 21 d after inoculation at 4 °C (*n* = 3). Infectious SARS-CoV-2 was quantified by plaque assay on Vero E6 cells and shown as log PFU/mL. The inoculum is shown as a solid gray line (3.9 log PFU/mL) and the detection limit of the plaque assay is shown as a dashed line (0.7 log PFU/mL). Significant differences between means are indicated by asterisks (** < 0.01; *** < 0.001).

From tomatoes and grapes, virus recovery remained constant through 7 dpi (*p* > 0.05), but infectious virus was significantly reduced by 14 dpi (3.5 and 3.6 log PFU/mL at 0 h to 1.3 and 1.1 log PFU/mL at 14 d, respectively, *p* < 0.05) (Figure 2). Nonetheless, more than 1 log PFU/mL of infectious virus could still be recovered from both tomatoes and grapes after 21 days in refrigeration conditions (4 °C). The initial virus recovery from the avocado shell at 0 h was 1.5–1.7 log PFU/mL lower than the other fresh produce and significantly less than the concentration of virus inoculated onto the samples (*p* < 0.05). However, infectious virus then remained relatively constant for 24 h. By 7 dpi, the virus was below the level of detection and completely undetectable by 14 dpi. In comparison, from the avocado pulp, the recovery of infectious virus was significantly reduced within 1 h and below the level of detection by 7 dpi (*p* < 0.05) (Figure 2).

### 3.3. Survival of SARS-CoV-2 on Meats

For meats, we selected meat cuts (beef steak and pork chop), as well as ground meats (beef and pork). We also tested a plant-based meat alternative since we have observed variable results in virus survival from fresh produce (in this study and previously) [29]. Although we previously assessed virus survival on shrimp and salmon [29], we included oysters in our current studies since they are often consumed raw.

**Figure 3:**
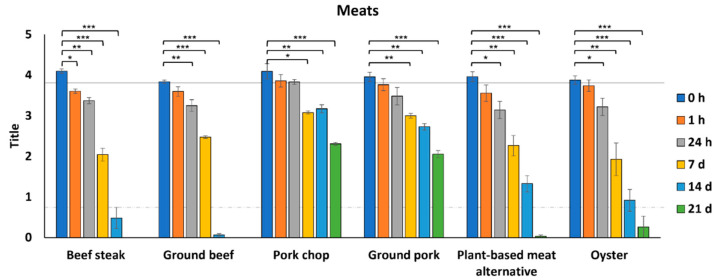
Survival of SARS-CoV-2 on meats. SARS-CoV-2 was recovered from foods immediately after inoculation (0 h) and at 1 h, 24 h, 7 d, 14 d, and 21 d after inoculation at 4 °C (*n* = 3). Infectious SARS-CoV-2 was quantified by plaque assay on Vero E6 cells and shown as log PFU/mL. The inoculum is shown as a solid gray line (3.9 log PFU/mL) and the detection limit of the plaque assay is shown as a dashed line (0.7 log PFU/mL). Significant differences between means are indicated by asterisks (* < 0.05; ** < 0.01; *** < 0.001).

In general, the various meats supported the survival of SARS-CoV-2 for more than two weeks at 4 °C (Figure 3). Although virus concentrations from all six meats were relatively similar for the first 7 days, we observed some differences by 14 dpi. Infectious SARS-CoV-2 decreased to 2.0 log PFU/mL on beef steak and 2.5 log PFU/mL on ground beef by 7 dpi and was below the level of detection on both beef steak and ground beef by 14 dpi. In contrast, infectious virus was recoverable from pork, plant-based meat alternative, and oysters at 14 dpi. Infectious virus was still recoverable from both pork chops and ground pork at 21 dpi, with a reduction of only 1.8–1.9 log PFU/mL of the original inoculum placed on the meat.

### 3.4. Survival of SARS-CoV-2 on Cooked Ground Beef

**Figure 4:**
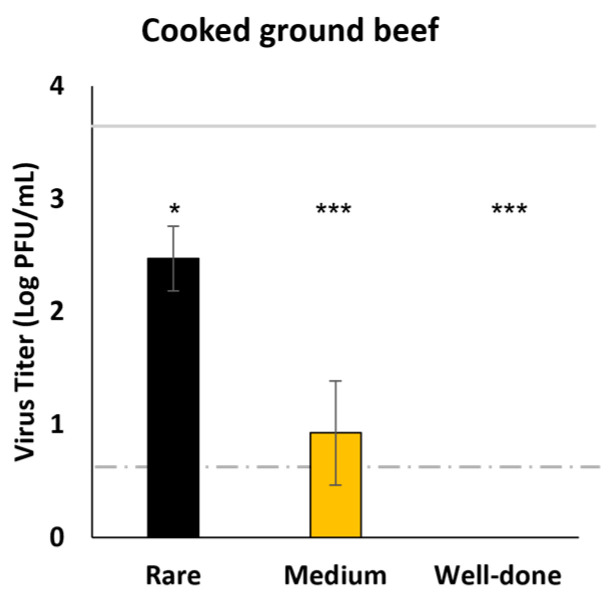
Survival of SARS-CoV-2 in ground beef burgers cooked to rare (125 °F/51.2 °C), medium (145 °F/62.5 °C), and well-done (160 °F/71.1 °C). Infectious SARS-CoV-2 was quantified by plaque assay on Vero E6 cells and shown as log PFU/mL. The inoculum is shown as a solid gray line (3.6 log PFU/mL) and the detection limit of the plaque assay is shown as a dashed line (0.7 log PFU/mL). Significant differences were compared between viral titers of cooked ground beef (rare, medium, or well-done) and uncooked ground beef (positive control), indicated by asterisks (* < 0.05; *** < 0.001).

Ground pork and plant-based meat alternatives are typically cooked well-done prior to consumption. However, ground beef formed into patties are commonly cooked to internal temperatures for rare (125 °F/51.2 °C), medium (145 °F/62.5 °C), or well-done (160 °F/71.1 °C) hamburgers based on the consumer’s preference. To determine if these internal temperatures could heat-inactivate SARS-CoV-2 in the matrix of ground beef formed into patties, we inoculated raw ground beef with the virus, formed 1.25 cm thick patties, and cooked them to internal temperatures for rare, medium, and well-done. Cooking times in a clamshell-style electric grill were approximately 1 min, 1.5 min, and 2 min for rare, medium, and well-done, respectively. Although no viable virus was detected in well-done beef patties, infectious virus was recovered from patties cooked to internal temperatures for rare (2.5 log PFU/mL) and medium (0.9 log PFU/mL), indicating that consuming rare and medium ground beef burgers may have potential risks of infection (Figure 4).

### 3.5. Relationship between Infectious Virus Titer and Viral Genome Copy Number

**Figure 5:**
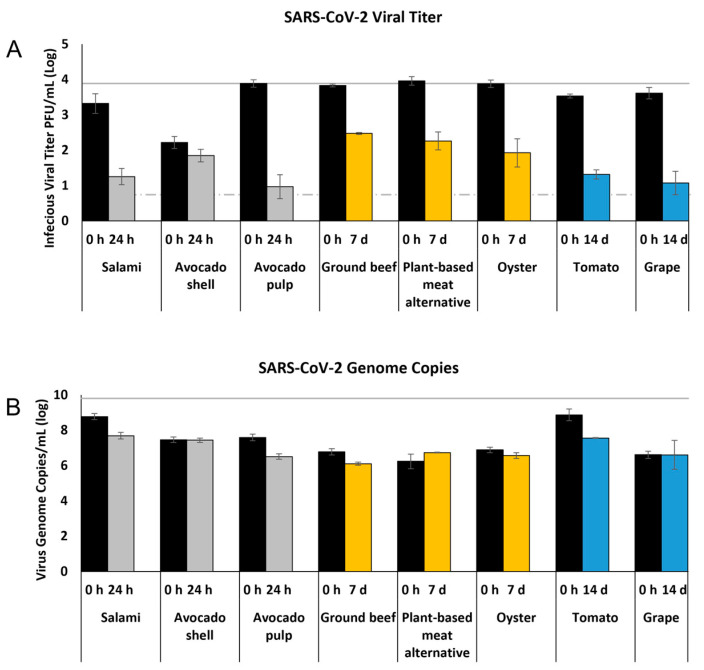
Comparison of SARS-CoV-2 infectious viral titer and viral genome copy number. (**A**) The titers for infectious SARS-CoV-2, as determined by plaque assay on Vero E6 cells, shown as log PFU/mL. (**B**) Viral genome copies of SARS-CoV-2, as determined by qRT-PCR using primers and probes specifically targeting SARS-CoV-2 nucleocapsid gene, shown as log viral genome copies/mL. Bar colors represent different holding times at 4 °C: black (0 h), gray (24 h), yellow (7 d), blue (14 d). The inoculum is shown as a solid gray line (3.9 log PFU/mL for viral titer or 3 × 10^9^ copies/mL for viral genome copy number) and the detection limit of the plaque assay is shown as a dashed line (0.7 log PFU/mL).

Pathogens are commonly detected and identified on foods and food packaging by swab collection, nucleic acid extraction, and PCR-based assays. To assess the relationship between infectious virus titer and viral genome copies for SARS-CoV-2 following virus recovery, eight of the foods were selected to represent the three food categories (deli, produce and meats) and qRT-PCR assay was conducted to determine the viral genome copy number (Figure 5). For each food, the qRT-PCR assay was conducted from two incubation time points: 0 h (initial recovery from food) and the incubation time point that had significant reductions in virus titer (>1 log in reduction) compared with 0 h. This time point was 24 h for avocado pulp; 7 d for ground beef, plant-based meat alternative, and oyster; and 14 d for tomato and grape. For avocado shell, which had a significant reduction in infectious viral titer compared to the inoculum dose at 0 h, we selected 0 h and 24 h for comparison with viral genome copy numbers.

In general, at 0 h the SARS-CoV-2 RNA copy number (Figure 5B) is 2–3 times higher than the infectious viral titer (Figure 5A) (6.2–8.9 log genome copies/mL vs. 2.2–4.0 log PFU/mL). At 24 h (salami, avocado pulp), 7 d (ground beef, plant-based meat alternative, and oyster), and 14 d (tomato and grape), infectious viral titers were significantly reduced compared to 0 h titers for each food (Figure 5A). However, genome copy numbers were not reduced at these same time points (Figure 5B). For the avocado shell at 0 h, we observed lower infectious viral titers at 0 h compared with avocado pulp (2.2 log PFU/mL vs. 3.9 log PFU/mL). However, avocado shell and pulp have similar viral genome copy numbers at 0 h (7.5 log copies/mL vs. 7.6 log copies/mL) and at 24 h (7.4 log copies/mL vs. 6.5 log copies/mL), indicating that RNA genome copy number is irrelevant to the infectious viral titer (Figure 5A,B).

## 4. Discussion

One of the most critical aspects of any pathogen is its ability to transmit and spread among populations [35]. Effective public health policies and mitigation strategies are reliant on a thorough understanding of transmission routes and potential fomites or vectors that may contribute to further spread, disease cases, and maintenance of the pathogen in human or animal reservoirs [35]. Although SARS-CoV-2 is known to be transmitted person-to-person by airborne transmission and respiratory droplets, other potential routes of infection have not been adequately investigated [4,11,12,30,36]. Although it is unlikely to be a major route of transmission, foodborne SARS-CoV-2 has the potential for entry into a host through ingestion, as the oral cavity and GI tract express the receptor and accessory protease by which the virus enters cells [18,19]. Here, we determined that SARS-CoV-2 can survive in an infectious state on several different types of foods, many of which are ready-to-eat or consumed raw, while other types of foods inactivate the virus and present a much lower risk of infection.

Following infection, COVID-19 patients may be asymptomatic or present with an array of variable symptoms. In addition to respiratory symptoms, gastrointestinal (GI) manifestations are common, including abdominal pain, vomiting, nausea, and diarrhea, sometimes prior to or independent of respiratory symptoms [37,38,39]. The angiotensin-converting enzyme (ACE2) receptor is abundantly expressed in the oronasal mucosa and esophagus, as well as in stomach and intestinal tissues [40,41], providing potential entry points for SARS-CoV-2 besides the respiratory tract. Additionally, several other molecules have been suggested as alternative receptors for SARS-CoV-2, including C-type lectins, TIM1 (T cell transmembrane, immunoglobulin, and mucin), AXL (AXL receptor tyrosine kinase), and NRP1 (neuropilin 1), suggesting that the virus may enter cells that do not express ACE2, as well [42]. Although no specific foodborne cases have been reported, outbreaks have been traced to imported foods or food packaging [26,27,43]. The mechanism of GI tract infection is not fully understood, but SARS-CoV-2 has been reported to infect the oral cavity, salivary glands, enterocytes and intestinal organoids, resulting in inflammatory cell recruitment and GI tissue damage [13,19,20]. In addition, intragastric inoculation in nonhuman primates led to the detection of the virus and inflammation in multiple tissues, including digestive tissues, lung, liver, and pancreatic tissues [20]. Although the acidity of the stomach (less than pH 3.5) typically inactivates most enveloped viruses [44], gastric pH can elevate to near neutral with a meal [45], which would allow viruses to survive transit through the stomach and cause infection in the intestines. MERS-CoV and HCoV-229E, two coronaviruses similar to SARS-CoV-2, were completely inactivated within 30 min in simulated fasting-state gastric fluid in vitro. However, they remained unaffected after 2 h of exposure in simulated fed-state gastric fluid, demonstrating that coronaviruses can likely survive the acidic environment of the stomach when food is also present [46]. Although SARS-CoV-2 was rapidly inactivated in simulated fasting-state gastric fluid in vitro [47], the stomach environment varies with the presence of a meal, which may allow SARS-CoV-2 to transit through the stomach and infect intestinal cells. Since SARS-CoV-2 can infect cells of the alimentary tract, consumption of virus-contaminated raw, undercooked, and ready-to-eat foods may be an alternative route for SARS-CoV-2 infection. Whether some types of foods pose a greater risk of infection is a question we sought to investigate.

In general, high-protein unprocessed and minimally processed foods (raw meat and seafood, roasted and chilled turkey) and foods high in both protein and fats (cheese and plant-based meat alternative) supported viable SARS-CoV-2 for at least 14 days at refrigeration temperature. In addition, foods with higher moisture content prolonged infectivity of SARS-CoV-2, likely by preventing their desiccation and inactivation. These findings concur with other studies reporting similar results [28,29]. SARS-CoV-2 on pork, beef, and salmon were reported to remain infectious for at least 9 days at 4 °C and 20 days at −20 °C [28]. Foods cannot support the replication of viruses, yet most foods cannot inactivate the virus. Ready-to-eat deli foods are often consumed without further cooking and oysters are commonly consumed raw. Since viruses may survive acidic gastric environments with a meal, consuming foods heavily contaminated with SARS-CoV-2 may produce a risk of transmission via GI tract infection. Beef, pork, and plant-based meat alternatives are usually cooked before consumption, effectively negating any risk of infection through ingestion. Previous studies have shown that SARS-CoV-2 is susceptible to heat inactivation in virus transport medium when heated to 70 °C (158 °F) for 5 min or 98 °C (208 °F) for 2 min [48,49]. SARS-CoV-2 spiked into human milk (7 log PFU) could also be inactivated by pasteurization at 62.5 °C (145 °F), but not at 56 °C (132 °F), for 30 min [50]. To ensure food safety, the USDA recommends steaks and roasts be cooked to the internal temperature of 62.5 °C (145 °F, medium), and ground beef and pork should be cooked to a minimum internal temperature of 71 °C (160 °F, well-done). Given that SARS-CoV-2 would be attached on the surface of steaks and roasts, cooking to an internal temperature of 145 °F should be sufficient to elevate the temperature of food surfaces higher than 158 °F, which would inactivate the virus on the food surface within a short time. However, ground beef, ground pork and plant-based meat alternatives could be contaminated by the virus within the matrix of the meat during grinding or preparation of burgers. Although ground pork and plant-based alternative burgers are typically cooked well-done, ground beef burgers are commonly cooked rare to medium (125 °F/51.2 °C to 145 °F/62.5 °C internal temperature), which requires a short period of cooking time and provides an environment rich in protein and fats conducive to survival of SARS-CoV-2. Our results demonstrated that SARS-CoV-2 survived in ground beef burgers cooked rare and medium, while no viable virus was detected in well-done ground beef burgers. Therefore, consumption of undercooked ground beef that has been contaminated with a high concentration of SARS-CoV-2 could be a potential risk for SARS-CoV-2 infection of the GI tract. Our findings highlight the importance of appropriate cooking, in addition to proper handling and infection control measures, to ensure food safety.

Although SARS-CoV-2 remained infectious on deli turkey and cheese for 21 days, virus concentration on hard salami was significantly reduced within 24 h post-inoculation. Hard salami is a dry-fermented sausage with a final pH of 4.8–5.3 (0.5–1% lactic acid) and with a moisture: protein ratio less than 2.3:1 in the end product [51]. Although the relatively dry environment on salami could contribute to the inactivation of a viable virus, the acidity may not, since a previous study reported that SARS-CoV-2 is not susceptible to inactivation in a wide range of pH values (pH 3–10) at room temperature [48]. In addition, food preservatives and flavor enhancers are usually added to processed meats to extend freshness and protect flavor. Butylated hydroxyanisole (BHA) and butylated hydroxytoluene (BHT) are added to salami to serve as antioxidants and antimicrobials to enhance food safety. BHT is reported to inactivate some enveloped viruses, including herpes simplex virus (HSV) and Newcastle disease virus [52]. Furthermore, citric acid is also added to salami to enhance flavor, and lactic acid is produced during the fermentation process. Organic acids, such as citric and lactic acid, are listed as active ingredients of SARS-CoV-2 disinfectants authorized by the Environmental Protection Agency in the United States [53]. Therefore, those organic acids present in salami could have antiviral effects against SARS-CoV-2. Collectively, inactivation of SARS-CoV-2 on salami could be caused by the comprehensive effects of low moisture, antioxidant additives, and organic acids produced during fermentation.

As we reported previously, fresh produce has variable effects on SARS-CoV-2 [29]. In our previous studies, infectious SARS-CoV-2 recovered from spinach and apple skin remained constant over 24 h post-inoculation, but mushrooms exhibited significant antiviral effects within one hour, destroying both infectivity and viral RNA. In this study, we determined that SARS-CoV-2 can survive with no substantial reduction for 7 days when inoculated onto the surface of tomatoes or grapes. After this time point, we observed a significant reduction in virus titer but viable SARS-CoV-2 was still detectable on tomato and grape outer skin 21 days after inoculation. Grape skin contains multiple phenolic compounds, such as resveratrol and gallic acid [54,55], which have previously been shown to inactivate viruses by binding of phenolics to the protein coat of the virus, interfering with the ability of the virus to bind to host cells [56]. In addition, grape extracts (skin and whole grapes) were reported to inactivate various enteric viruses and HSV-1 [57], and grape pomace exhibited antiviral effects against adenovirus [58] and influenza [59]. However, our results demonstrated that virus concentration on the surface of intact grapes was not significantly reduced within 7 days, indicating that the outer grape skin did not have substantial antiviral effects against SARS-CoV-2. As cherry tomatoes and grapes are typically eaten raw, these results highlight the importance of washing fresh produce prior to consumption. Anecdotally, people who are concerned about SARS-CoV-2 contamination of foods have been known to soak their produce in bleach, which can introduce dangerous levels of sodium hypochlorite into fruits with porous skins, such as tomatoes and grapes. Our method of virus recovery from foods, in which rinsing with media recovers nearly 100% of inoculated virus from the surface of the foods immediately after inoculation, suggests that simply rinsing produce with water is sufficient to remove nearly all infectious virus.

Compared to tomatoes and grapes, we recovered a less viable virus from both the outside (shell) and inside (pulp) of avocados. Additionally, initial virus recovery (0 h) from the avocado shell was substantially less than the other produce. The avocados used in these studies were purchased from the local grocery store and avocados are often coated by commercial antimicrobial sprays, potentially contributing to the lower initial virus recovery. In contrast to tomatoes and grapes, avocado pulp exhibited a strong antiviral effect against SARS-CoV-2, reducing infectious virus to less than 1 log PFU/mL within 24 h. Extracts of avocado pulp exhibit antiviral activity against dengue virus by inhibiting viral replication, causing an increased survival rate among mice infected with the virus [60]. Other studies have reported that phenols and unsaturated fatty acids have antiviral effects against SARS-CoV-2. Rutin, one of the main flavanols in ripe avocado pulp [61], was found to have high antiviral activity as a SARS-CoV-2 protease inhibitor, preventing viral replication [62]. However, the mechanism of antiviral activity must be different with SARS-CoV-2 placed directly on avocado pulp, as the virus does not replicate on the foods. The flavonoids quercetin and luteolin, as well as the polyphenol gallic acid, in avocado pulp show high binding affinity towards the ACE2 receptor in human cells, potentially inhibiting SARS-CoV-2 attachment to ACE2 [63,64]. In addition, the free fatty acid linoleic acid, which comprises 19.3% of pulp oil from the Barker cultivar [65], tightly binds to the receptor binding domain of SARS-CoV-2, resulting in the reduction in interaction between the ACE2 receptor of human cells and the spike protein of SARS-CoV-2 [66]. Thus, the avocado pulp contains a multitude of phytochemicals that may inhibit the binding of SARS-CoV-2 to its receptor ACE2, effectively reducing the infectivity of the virus.

In the food industry, the gold standard for foodborne pathogen detection is based on rapid PCR screening [67]. For bacteria, PCR-based detection is confirmed by growth in appropriate media [67]. Confirmation of foodborne viruses is challenging, however, due to a lack of necessary facilities, expertise, and time associated with propagating a virus in an appropriate cell culture, particularly for viruses that may require higher biocontainment environments. Although nucleic acid extraction and PCR-based assays are standardized for the common foodborne viruses hepatitis A and norovirus, confirmatory assays to demonstrate a viable virus are rarely performed [68]. The detected presence of the viral genome typically initiates disinfection procedures and disposal of presumably contaminated foods, which can become quite costly. To determine if a PCR-based assay for SARS-CoV-2 would accurately correlate with the quantity of infectious virus, we investigated the relationship between viral RNA genome copy number, using a PCR-based assay, and infectious viral titers, determined by plaque assay, in several foods from our three categories (deli, produce, and meats). At all time points tested, SARS-CoV-2 viral RNA copies were 2–3 times higher than infectious viral titers at the same time point. Regardless of food type, infectious SARS-CoV-2 was inactivated over time, while viral RNA was not degraded in similar trends. Therefore, the presence of viral RNA identified by PCR-based tests is not necessarily equivalent to the presence of infectious virus in foods. Without a confirmatory test to demonstrate the presence of infectious virus, costly disinfection procedures and disposal of potentially contaminated foods may be conducted based solely on a PCR-based assay that cannot differentiate between the presence of non-infectious viral genome fragments and a viable virus that may pose a risk of infection. SARS-CoV-2 RNA has previously been detected on food and food packages, based on PCR-based assays [26,27,69]. However, previous studies, including ours, have also reported that SARS-CoV-2 RNA can be present on food, while infectious SARS-CoV-2 is undetectable by plaque assay [29,70]. Recent studies have reported on the development of amplification-free CRISPR-Cas assays for direct detection of SARS-CoV-2 RNA from patients as alternatives to PCR-based assays [71,72,73]. Although CRISPR-based assays produce results more rapidly than PCR amplification-based assays, they still detect viral RNA but not viral infectivity. PCR-based tests or CRISPR-based assays may be used for an initial screen, but the presence of an infectious virus must be confirmed by other methods, since the infectious virus is the form that presents the risk to public health. Rapid and inexpensive technologies are thus needed in the food industry to reliably confirm the presence of infectious foodborne viruses to avoid economic losses from disposal of food thought to be contaminated, as well as from costly disinfection procedures that may not be warranted.

## 5. Conclusions

Taken together with our previous study [29], we assessed the survival of SARS-CoV-2 on a variety of different types of foods. Although previous studies primarily focused on investigating the survival of SARS-CoV-2 on cold-chain meats and seafoods, this is the first assessment of the survival of SARS-CoV-2 on broad categories of foods, including produce, deli foods, meats and seafoods, which is of interest to the food industry as well as consumers. Meats, seafoods, and deli foods that are high in protein, fats, and moisture maintain infectivity of SARS-CoV-2 for up to three weeks when the foods are held at refrigeration temperature (4 °C). However, food additives and antioxidants naturally found in various types of fresh produce contribute to inactivation of infectious virus. We highlight the importance of rinsing produce that will be consumed raw and properly cooking foods to inactivate any contaminating virus with heat. Although SARS-CoV-2 can remain infectious on a variety of different types of foods, further studies are needed to determine whether ingestion of virus-contaminated foods would result in infection and how symptoms following ingestion may differ from respiratory transmission.

## Figures and Tables

**Table 1:** Foods tested in this study, grouped into three broad categories: deli, produce, and meats.

Foods	Specific Cuts and Information
Deli	
Turkey	Oven-roasted turkey breast, sliced
Cheese	Swiss cheese, sliced
Salami	Hard salami, sliced
Produce	
Tomato	Cherry tomato, entire fruit, not cut
Avocado shell	Ripe avocado outer shell
Avocado pulp	Ripe avocado pulp
Grape	Red grape, entire fruit, not cut
Meats	
Steak	Choice top round steak
Ground beef	80% lean and 20% fat
Pork chop	Center cut chop
Ground pork	72% lean and 28% fat
Plant-based meat alternative	Plant-based pre-formed patty
Oyster	Fresh oyster removed from shell

## Data Availability

Data are available upon request.
